# Efficacy, safety and cost-effectiveness of insulin sensitizers as add-on therapy in metabolic syndrome in patients with secondary sulfonylurea failure: A comparative study

**DOI:** 10.4103/0976-500X.72349

**Published:** 2010

**Authors:** Rajiv Mahajan, Anita Gupta, Rajinder Singh Gupta, Kapil Gupta

**Affiliations:** *Department of Pharmacology, Adesh Institute of Medical Sciences and Research, Bathinda - 151 109, Punjab, India*; 1 *Department of Pharmacology, Government Medical College, Patiala - 147 001, Punjab, India*; 2 *Department of General Medicine, Government Medical College, Patiala - 147 001, Punjab, India*; 3 *Department of Biochemistry, Adesh Institute of Medical Sciences and Research, Bathinda - 151 109, Punjab, India*

**Keywords:** Body mass index, insulin resistance, metformin, pioglitazone

## Abstract

**Objectives:**

Prevalence of metabolic syndrome (MS) is ~25% and it is currently becoming prevalent in children also. India is estimated to have the maximum number of patients of MS in the world. As insulin resistance is an integral part of MS and the rate of secondary sulfonylurea failure (SSUF) is also high, the present study was planned to evaluate the effects of insulin sensitizers as add-on therapy in MS with SSUF.

**Materials and Methods:**

An open-label, prospective, randomized study was conducted on 200 patients of MS with SSUF, included according to ATP III criteria, after dividing them into two groups. Group I patients were given pioglitazone 30 mg/day while group II patients were given metformin 1,000 mg/day as add-on therapy to the sulfonylurea already prescribed.

**Results:**

Fall in fasting blood glucose, glycosylated hemoglobin and serum triglycerides was higher with metformin, but rise in high-density lipoprotein–cholesterol was higher with pioglitazone. Only metformin caused a significant reduction in body mass index. Significant reduction in waist circumference, systolic blood pressure and diastolic blood pressure was not seen with any therapy. Incremental cost-effective ratio was almost six-times higher with pioglitazone.

**Conclusion:**

Among insulin sensitizers, metformin has more favorable, persistent and multifacet effects in MS with SSUF. Studies of longer duration are required for calculating reduction in the mortality and morbidity.

## INTRODUCTION

Metabolic syndrome (MS), also known as insulin resistance syndrome (IRS) or Syndrome X, is a collection of risk factors that include insulin resistance, central obesity, arterial hypertension and atherogenic dyslipidemia in type-2 diabetic patients and carries increased risk for cardiovascular disease.[1] The term metabolic syndrome was used for the first time by Haller in 1977.[2] It affects one in five people and the prevalence increases with age. Some studies estimate the prevalence in the USA to be up to 25% of the population.[3] The prevalence of MS is high among European obese children (12.2%) and this rapid rising prevalence of childhood obesity is related to an increased risk of obesity-related diseases during adulthood.[4] Given that India has the largest number of subjects with type-2 diabetes in the world, it can be extrapolated that this country also has the largest number of patients with the metabolic syndrome. Epidemiological studies confirm a high prevalence.[5]

The two major therapeutic strategies for the treatment of affected persons are prevention by modification of the underlying risk factors and separate drug treatment of the particular metabolic risk factors when appropriate.[6] Drug therapy is needed to achieve recommended goals if therapeutic lifestyle changes are not sufficient. As the cause of MS is multifactorial, treatment should also be multifacet. At present, the drugs used in MS are antiobesity drugs like sibutramine and rimonabant, antidiabetic drugs like sulfonylureas (SU), metformin and pioglitazone and statins and fibric acid derivatives.

Type-2 diabetes is an integral part of MS. The SU class of antihyperglycemic medications has been used as oral therapy for type-2 diabetes since 1954.[7] But, a few relatively recent reports have described secondary SU failure (SSUF). The United Kingdom Prospective Diabetes Study found that 50% of normal and overweight patients failed to maintain glycosylated hemoglobin (HbA_1C_) <7% after 3 years.[8] Boccuzzi *et al*. reported secondary failure rates of 21.8% during a 12-month follow-up.[9] Another study in Saskatchewan reported the addition or switch of antihyperglycemic drugs in 46.8% of the patients who had used SU agent for at least 2 years.[10] A British study found that SU failure began as quickly as 6 months after initiation when added to metformin as a second-line therapy.[11]

Metformin and pioglitazone are oral antidiabetic drugs that are known to improve insulin resistance. Both these drugs improve the sensitivity of the peripheral organs to insulin thus improving glucose control. Additionally, they have beneficial effects on the lipid profile.[6] Accordingly, the present study was designed to evaluate the efficacy, safety and cost-effectiveness of insulin sensitizers, metformin and pioglitazone, as add-on therapy in patients with MS with SSUF.

## MATERIALS AND METHODS

The present study was a 24-week open-label, prospective, randomized, parallel-group, active treatment-controlled, single-dose, add-on designed trial with intent to treat. The study was approved by the Institutional Ethical Committee and was conducted according to the International Conference on Harmonization-Good Clinical Practice (ICH-GCP) guidelines and the guidelines issued by the Indian Council of Medical Research, 2006. Two hundred adult patients diagnosed with MS according to Adult Treatment Panel III (ATP III) criteria,[12] and having SSUF, attending the Medicine Out-Patient Department and Diabetic Clinic, were included in the study. SSUF was defined in two ways: (1) the addition of or switch to another antihyperglycemic drug after 6 months of treatment with SU or (2) the first HbA_1C_ measurement ≥64 mmol/mol (or 8%) that occurred before the addition or switch of antihyperglycemic therapy.[13] Accordingly, the inclusion and exclusion criteria were as follows.

### Inclusion criteria

Secondary sulfonylurea failure patients (defined by any one of the two criteria described above) having any three of the following five features were included in the study.

- Waist circumference (WC) >40 inches or body mass index (BMI) >27 kg/m^2^ in men and WC >35 inches or BMI >25 kg/m^2^ in women.
- Serum triglycerides (TGs) ≥150 mg/dl.
- High-density lipoprotein-cholesterol (HDL-C) <40 mg/dl in men and <50 mg/dl in women.
- Blood pressure ≥130/≥85 mmHg.
- Fasting blood glucose (FBG) ≥110 mg/dl.

### Exclusion criteria

- Patients with clinical sign and symptoms of type-1 diabetes.
- Patients with diabetic retinopathy, neuropathy or nephropathy.
- Patients with impaired liver function tests, i.e. serum glutamic oxaloacetic transaminase (SGOT) and/or serum glutamic pyruvic transaminase (SGPT) ≥100 U.
- Pregnant or lactating women.
- Patients taking medications that could affect blood glucose metabolism, i.e. patients on non-selective β-blockers, diuretics and corticosteroids.

Patients were randomly divided into two groups of 100 each, using a randomized card system, after taking a duly informed written consent. Group I patients were given 30 mg pioglitazone (Tab. Glizone; Cadila, Ahmedabad, India) once daily while Group II patients were given 500 mg metformin (Tab. Insumet; Cadila, Ahmedabad, India) two times a day as add-on therapy to the SU already prescribed. Type and dose of SU already taken by patients was not changed. Provision was made to add another drug where required, but not to increase the dose of insulin sensitizers. As ours was an intent-to-treat study, even dropout cases were considered to have completed the study and were included in the final analysis.

Efficacy was measured by measuring the effect on surrogate end points that include: FBG, HbA_1C,_ TGs, HDL-C, WC, BMI, systolic blood pressure (SBP) and diastolic blood pressure (DBP). Safety was measured by noting episodes of hypoglycemia (if any) or other adverse effects of the drugs. For calculating cost-effectiveness, direct costs of medications and favorable significant effect on all surrogate end points were taken into consideration. For calculating effective outcomes, each unit favorable significant change (increase or decrease) in all parameters was assigned a value of one, irrespective of the parameter or surrogate end point, while non-significant effects were ignored for analysis. All parameters were measured at the start of therapy, at 12 weeks and then at the end of therapy (after 24 weeks), except FBG, which was measured weekly. For analysis, the values of all surrogate end points at the end of the therapy (at 24 weeks) were compared with the baseline values. Paired *t*-test was used for statistical analysis.

## RESULTS

Age of participants varied from 36 to 67 years. Mean age was 49.16 ± 5.90 and 49.23 ± 5.90 years in Groups I and II, respectively and this difference was statistically non-significant. There was a statistically non-significant preponderance of males in both groups.

Fall in mean FBG, HbA_1C_ and TGs levels and rise in mean HDL-C level was highly significant (*P* < 0.001) with both therapies after the completion of treatment as compared to baseline. Only addition of metformin had a significant effect in lowering BMI, while pioglitazone had a non-significant effect on BMI in SSUF patients of MS and both therapies failed to show any significant reduction in WC. A non-significant (*P* > 0.05) effect on SBP and DBP was seen with both therapies [Table 1].

**Table 1:** Effect of add-on therapy of pioglitazone and metformin on surrogate end points in MS patients with SSUF

Parameter (units)	With pioglitazone add-on therapy		With metformin add-on therapy	
	Before treatment	After treatment	Before treatment	After treatment
FBG (mg/dl)	150.41 ± 11.56	118.95 ± 7.24*	158.91 ± 10.12	110.01 ± 7.84*
HbA_1c_(mmol/mol)[14]	68.54 ± 9.72	52.14 ± 8.12*	71.53 ± 10.36	50.80 ± 8.78*
TGs (mg/dl)	202.71 ± 18.05	177.48 ± 19.16*	205.38 ± 16.67	160.34 ± 13.37*
HDL-C (mg/dl)	40.20 ± 4.10	45.43 ± 3.98*	0.47 ± 3.19	43.82 ± 3.32*
WC (inches)	41.05 ± 4.67	40.97 ± 4.73	41.83 ± 5.26	41.28 ± 4.96
BMI (kg/m^2^)	28.84 ± 1.57	28.96 ± 1.53	28.92 ± 1.87	26.43 ± 1.32*
SBP (mmHg)	141.73 ± 10.31	140.49 ± 9.19	142.67 ± 9.39	140.33 ± 8.29
DBP (mmHg)	84.33 ± 7.52	83.27 ± 5.27	84.80 ± 5.55	83.53 ± 3.81

All values are mean ±SD. n=100 in each group.

* *P*<0.001 significantly different from baseline. MS- metabolic syndrome; SSUF- Secondary sulfonylurea failure.

On comparing the mean changes in different surrogate end points at the end of therapy as compared to baseline (pre-treatment), more fall in FBG, HbA_1C_ and TGs levels was documented with metformin add-on therapy as compared to pioglitazone (30.76% vs. 20.91%, 28.98 vs. 23.92% and 21.93% vs. 12.47%, respectively) and this difference was highly significant (*P* < 0.001). Increase in the HDL-C level was significantly higher with pioglitazone add-on therapy as compared to metformin (13.01% vs. 8.27%). Fall in BMI was significantly higher with metformin add-on therapy as compared to pioglitazone add-on therapy in patients of MS with SSUF. For all other surrogate end points, the comparative difference between the two therapies was non-significant [Table 2].

**Table 2:** Comparative change in surrogate end points at the end of the study as compared to pre-treatment values with add-on therapy of pioglitazone (Group I) and metformin (Group II) in MS patients with SSUF

Parameter (units)	Mean change (from baseline)		Percentage change		Comparative mean change
	Group I	Group II	Group I	Group II	
FBG (mg/dl)	31.46 ± 5.24↓	48.90 ± 6.41↓	20.91	30.76	17.44 ± 5.43*
HbA_1C_ (mmol/mol)	16.40 ± 4.26↓	20.73 ± 4.79↓	23.92	28.98	4.33 ± 5.98*
TGs (mg/dl)	25.23 ± 10.32↓	45.04 ± 10.56↓	12.47	21.93	19.81 ± 9.34*
HDL-C (mg/dl)	5.23 ± 1.87↑	3.35 ± 1.19↑	13.01	8.27	1.88 ± 2.01*
WC (inches)	0.08 ± 4.06↓	0.56 ± 4.90↓	0.19	1.34	0.48 ± 3.46
BMI (kg/m^2^)	0.12 ± 0.92↑	2.49 ± 0.82↓	0.41	8.60	2.37 ± 1.03*
SBP (mmHg)	1.24 ± 1.56↓	2.33 ± 5.09↓	0.87	1.63	1.09 ± 2.43
DBP (mmHg)	1.07 ± 5.21↓	1.27 ± 4.15↓	1.26	1.49	0.20 ± 3.98

↓, decrease; ↑, increase. All values are mean ±SD. n=100 in each group.

* significantly different. MS- metabolic syndrome; SSUF- Secondary sulfonylurea failure.

At the end of therapy, in 36% of the patients treated with pioglitazone add-on therapy, FBG levels still remained above the recommended limit of 110 mg/dl. The corresponding figure in patients treated with metformin add-on therapy was 19%. Moreover, in patients treated with pioglitazone, although a highly significant statistical reduction in TGs was seen, the mean TGs levels remained close to the normal maximum limit of 180 mg/dl at the end of the study.

Pedal edema and headache were the most common adverse effects reported with pioglitazone (3%), while gastrointestinal disturbance was the most common adverse effect reported with metformin add-on therapy (5%). Only one episode of hypoglycemia was reported with metformin add-on therapy. One percent of the patients reported generalized weakness in both groups, while headache was reported in one patient on metformin add-on therapy. Withdrawal of therapy due to adverse effects was not necessitated in any patient. Only one patient on pioglitazone add-on therapy was shifted to insulin due to poor response at the 14^th^ week of treatment.

With pioglitazone add-on therapy, a significant favorable effect was observed in FBG, HbA_1C_, TGs and HDL levels while with metformin add-on therapy, an additional significant favorable effect was observed in BMI. After assigning a value of one to each unit significant favorable change in different parameters, the effective outcomes observed were 78.32 units with pioglitazone while they were 120.51 units with metformin add-on therapy. Therefore, the Incremental Cost Effective Ratio (ICER) observed was -6.72, clearly in favor of metformin [Table 3].

**Table 3:** Cost-effective analysis of pioglitazone and metformin therapies

Groups	Units of favorable significant change	Mean cost of treatment (Rs.)	ICER
Group I	78.32	553.40	-6.72
Group II	120.51	269.80	

ICER - Incremental cost effective ratio

## DISCUSSION

Metabolic syndrome is becoming a common disorder. Amelioration of insulin resistance early in the natural course of the disease is of utmost importance. Pioglitazone enhances insulin sensitivity in liver, adipose tissue and skeletal muscle by binding to nuclear peroxisome proliferator activated receptor-gamma (PPAR-γ), thereby regulating the expression of numerous genes, resulting in improved insulin-mediated glucose disposal and also modifies the atherogenic lipid profile without stimulating insulin secretion.[15] Metformin, a biguanide, lowers glucose levels by increasing tissue utilization of glucose or reduced absorption of glucose from the gastrointestinal tract without increasing insulin secretion.[16]

The results of the present study are comparable with earlier reported studies. Aronoff *et al*. had reported a mean fall in FBS levels by 18.1–55.9 mg/dl (6.87–20.29%), 8.9–9.3% decrease in TGs levels and an increase of 7.9–19.1% in HDL-C levels with pioglitazone 7.5–45 mg/day over a period of 26 weeks,[17] while in the present study, the corresponding figures with pioglitazone add-on therapy were 20.91%, 12.47% and 13.01%, respectively.

Goodman *et al*. reported a fall of 22% in FBG levels over a period of 6 months with metformin 850–2,250 mg/day[18] while Defronzo *et al*. reported a decrease by 45% in TGs and increase by 17% in HDL-C with metformin 2.5 g/day for 3 months.[19] In the present study, reductions in FBG and TGs levels were 30.76% and 21.93%, respectively, while increase in HDL-C was 8.27% with metformin add-on therapy. A lesser decrease in TGs levels and a lesser increase in HDL-C in the present study may be due to the lower dose of metformin used (1 g/day) as compared to earlier studies (up to 2.5 g/day).

With metformin therapy, a significant decrease of 8.6% was observed in BMI, but no such decrease was reflected in terms of reduction in WC. WC reduced non-significantly by 1.34% with metformin therapy. Ozata *et al*. reported a highly significant fall of 1.83% in BMI and 1.43% in WC with 3 months treatment of 1,750 mg/day of metformin.[20] A higher decrease in BMI with metformin in the present study may be due to the long treatment period of 6 months as compared to the 3-months treatment period in earlier studies. Although percentage reduction in WC with metformin therapy in the present study was comparable to that by Ozata *et al*., however, in the present study, this reduction was statistically non-significant and the study was not long enough to assess the clinical significance of this reduction in WC.

In the present study, increase in HDL-C was higher with pioglitazone add-on therapy, but fall in FBG and TGs was significantly more with metformin. Moreover, only metformin showed prominent fall in BMI. Thus, metformin produced multifacet actions in MS. Both drugs were fairly tolerable and safe. More persistent action of metformin on FBG and TGs, higher number of patients having controlled diabetes with metformin therapy, i.e. FBG levels <110 mg/dl (36% vs. 19%) and more favorable cost-effective ratio makes metformin a better option in MS as add-on therapy in SSUF patients. Studies of longer duration, with addition of antihypertensive agents and fibric acid derivatives, are required to fully establish the role of insulin sensitizers in MS and to derive a protocol for the treatment of MS.
